# Survey of the Factors Associated with a Woman's Choice to Have an Epidural for Labor Analgesia

**DOI:** 10.1155/2010/356789

**Published:** 2010-06-29

**Authors:** Jennifer Harkins, Brendan Carvalho, Amy Evers, Sachin Mehta, Edward T. Riley

**Affiliations:** ^1^University of South Florida College of Medicine, Tampa, FL 33612, USA; ^2^Department of Anesthesia, Stanford University School of Medicine, Stanford, CA 94305, USA

## Abstract

*Objectives*. The purpose of this study was to determine the factors associated with whether a woman received an epidural in labor and to determine the main source used to obtain information about labor epidurals. *Methods*. Over a one-month period, we surveyed all patients who labored, the day after their delivery. We used multiple logistic regression to identify potential predictive factors after initial univariate analysis. *Results*. 320 women who met enrollment criteria delivered during the study period and 94% completed the study. Of the 302 patients surveyed, 80% received an epidural for labor. Univariate analysis showed the following variables were associated with whether women received an epidural (*P* < .01): partner preference, prior epidural, language, education, type of insurance, age, duration, and pitocin use. Using computed multiple logistic regression only partner preference and prior epidural were associated with whether women received an epidural. *Conclusion*. It was not surprising that a previous epidural was predictive of a patient receiving an epidural. The strong association with partner preference and epidural use suggests this is an important factor when counseling pregnant women with regard to their decision to have a labor epidural.

## 1. Introduction

Epidural analgesia provides excellent pain relief and many parturients in the United States use this technique for analgesia during labor [1, 2]. The labor epidural use in the United States has increased dramatically over the past decade (from 22% to 61% of deliveries between 1981 and 2001) [1, 2]. Fifty one percent of parturients at hospitals performing at least 1,500 deliveries annually receive epidural analgesia. A recent survey of California hospitals found the median labor epidural rate to be 65% (Range 0–95%) [3]. In France, 75% of women receive epidural analgesia during labor [4, 5]. At our institution, (Lucile Packard Children's Hospital, Calif) approximately 80% of laboring women receive epidural analgesia. Although epidural analgesia provides effective pain relief in labor, there are women who choose not to use the technique during childbirth. Obstetric practices emphasize unmedicated births to varying degrees. Some obstetricians and midwives will emphasize nonpharmacologic pain relief and coping strategies and many women seek these practices out. The advantages of avoiding an epidural is that women may have a higher success breast-feeding, better mobility, and possible lower risk of instrumental vaginal delivery. Other reasons cited for avoidance of labor epidurals include fear of back pain and perceived increased risk of cesarean delivery [6–8]. 

Sources of information that women use to educate themselves about the procedure may also be an important factor in whether they request an epidural during labor. Studies have shown that women who receive labor epidurals are more likely to have attended childbirth classes or read related books [9]. In addition, factors extrinsic to patients may impact on whether women receive labor epidurals. Other reasons including anesthesia providers not available in a timely fashion, family members discouraging the patient, and previous patient experiences may influence future choices [10]. Practice policies at the hospital may also prevent a laboring patient from receiving an epidural.

The objectives of this study were to determine what factors are associated with whether a woman received an epidural for labor analgesia on our labor and delivery service. The specific aims were to

1. identify important factors associated with women receiving, or not receiving labor epidural analgesia,
2. determine the primary source of information concerning epidural analgesia used by women in our hospital.

## 2. Methods

After institutional review board approval, all the women who labored and delivered at Lucile Packard Children's Hospital between September 12, 2006 and October 12, 2006 were approached for the study survey. Women included in the study were those that were admitted for labor and went on to deliver vaginally or those whom required a cesarean delivery due to complications that occurred while attempting a vaginal delivery. Exclusion criteria included women who underwent planned cesarean deliveries, cesarean deliveries without prior admission for labor, multiple gestation pregnancies, and stillbirth deliveries. 

Women were requested to complete the survey following informed consent. The patients read the survey and study investigators were available to assist participants completing the survey and clarify any questions that arose. Verbal and written Spanish translation was provided to those whose first language was Spanish. The survey was distributed early in the morning or the early afternoon after delivery. 

The survey consisted of two sections. The first section examined desire for an epidural on a scale of 0–100 with questions geared towards the reasons for wanting or not wanting an epidural, the primary source of information on epidural analgesia, potential external influences (e.g., spouses or relatives), previous experience from a past delivery, and demographics (age, height, weight, ethnicity, language, religion, and education). These questions were developed from a focused discussion by the authors. The second section looked at information regarding the admission date, time, and day of week, whether the delivery was a scheduled or induced, if oxytocin was required, time of delivery, weight of baby, obstetric group, and insurance status. Each question expressed only one idea (i.e., no questions contained “and”) and no questions were phrased in a negative form. Answer types included choosing from a menu of choices, yes/no/neutral, or desire scores on a scale of 0 to 100 (0 = absolutely not and 100 = absolutely). The survey questions are all included in the appendix. Data, such as insurance type, mode of delivery, and pitocin use were obtained from a review of the chart.

### 2.1. Statistical Analysis

Calculation of the sample size was based on the number of factors we wanted to analyze as possible predictors of the use of epidural analgesia during labor. In order to evaluate up to fifteen predictive factors in a multivariate model and ensure stability of the regression calculation, we estimated the requirement of 10 patients with the outcome measure (whether a labor epidural was received) per degree of freedom. Thus, a minimum of 150 patients would be required. We calculated that we could easily enroll 150 patients in a one month period based on our institution's delivery rate and our approximately 80% epidural rate. We elected to enroll every patient for one full month to minimize fluctuations in weekday and weekend practices.

Descriptive statistics were used to summarize demographic and outcome. We first conducted univariate analyses (Student *t*-test, Wilcoxon-Mann-Whitney or Chi-squared tests as appropriate) to analyze factors determined *a priori* to be potentially important. A factor found to be significantly (*P* < .01) association with a patient having an epidural with univariant analysis was considered a potential predictive factor. These independent variables were then included in a multiple logistic regression analysis. A *P* < .05 was considered significant for multivariate analysis. The more rigorous *P*-value for the univariate analysis was used to partially account for the multiple comparisons. Data were analyzed using Microsoft Excel and SPSS (Version 11).

## 3. Results

**Figure 1:**
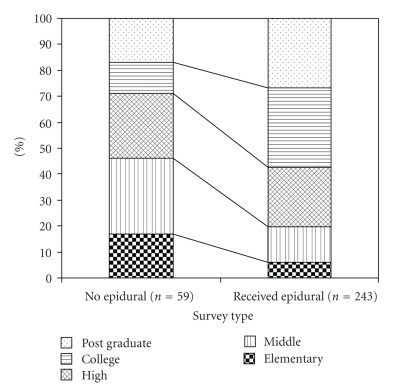
The highest educational level achieved by the women surveyed. Women receiving labor epidurals had a higher overall educational level (*P* < .05).

A total of 320 women who fit enrollment criteria delivered during the study period. Of these, 302 patients completed the survey. Eighty percent of the women received an epidural for labor during the study month. Demographic variables including age, height, weight, and BMI were all comparable between women that did or did not receive an epidural during labor (Table 1). Women that received an epidural had a stronger desire for an epidural prior to labor and delivery. These women were more likely to be nulliparous, to have had an epidural in the past, to speak English as their primary language, to have finished high school, and to have received more years of education (Table 2, Figure 1). Women that received an epidural were less likely to be Hispanic and have MediCal (California's equivalent of Medicaid) insurance, and their partners were less likely to object to them receiving an epidural during labor. In addition, their labors tended to be longer and they were more likely to receive pitocin compared to women who did not receive a labor epidural (Table 2, Figure 1). 

Utilizing univariate analysis we found the following factors to be independent predictors of epidural use (*P* < .01). Partner preference, prior epidural, English as primary language, level of education, type of insurance, age, duration of labor, and pitocin use. Combining these factors into a multiple logistic regression model, only partner preference and a prior epidural were identified to be associated with whether women received an epidural during labor (Table 2).

Of the 243 patients who received an epidural 53 (22%) did not want a labor epidural (as reflected by a desire for an epidural prior to the labor experience score of 0 on a scale of 0–100; 0 = absolutely not and 100 = absolutely) and 107 (44%) had an epidural desire score of 50 or less. All but one patient that had an epidural desire score of 100 (86 women) received an epidural. Of the women who did not receive an epidural (*n* = 59), 43 (73%) had a desire score of 0 and 55 (93%) had a desire score of 50 or less. 

Prior to labor, the primary reason why the total study population (*n* = 302) of patients in this survey may have wanted an epidural was “pain control” (77%), followed by “previous experience” (6%), and “encouraged to obtain an epidural by friends or family members” (6%). The reasons for receiving an epidural were not different between the women that had an epidural versus those that did not (Table 3). However, the groups differed in reasons why they wanted to avoid an epidural (Table 4). For women that actually *received *a labor epidural, pain relief was by far the number one reason (Table 5). In women that *did not receive* an epidural, the reasons for not obtaining an epidural were varied (Table 6) with concern of risk to themselves and a desire for natural childbirth being the main reasons.

**Figure 2:**
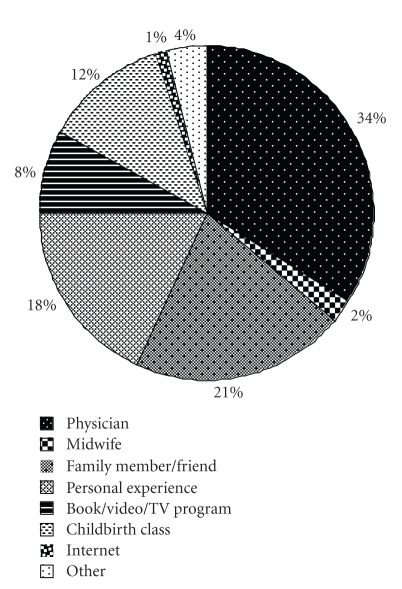
The main sources of information use by women surveyed (*n* = 301) to learn about epidurals prior to going into labor.

The sources from which women obtained their information about epidurals were varied (Figure 2). There were five important sources of information (the obstetrician, family and friend, childbirth classes, books, and previous experience). There were no correlations with the source of information and whether a patient received an epidural except that, as stated before, having received a previous epidural.

**Figure 3:**
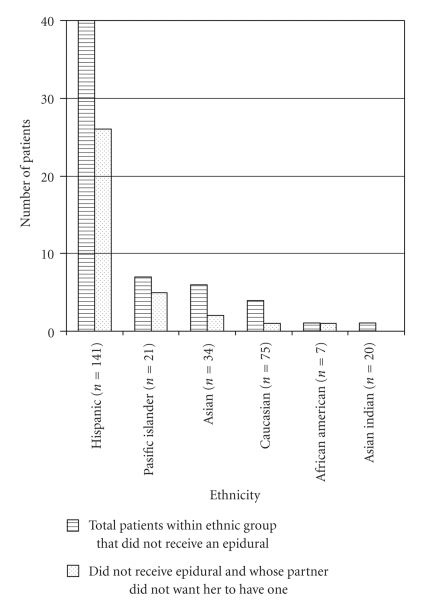
Ethnic group breakdown of women who did not receive epidurals and their partner preference for labor epidurals.

Hispanic women were the largest ethnic group delivering in the hospital and they represent a disproportionate number of women who did not receive epidurals (Table 1; Figure 3). This ethnic group appeared to have the largest influence from partner preference for an epidural.

## 4. Discussion

Partner preference and having had a previous epidural were the two factors that emerged from the multiple logistic analysis as significant predictors of a woman receiving an epidural during the month we studied our service. It is completely understandable that a parturient's previous experience would influence her next delivery. Although it may be remarkable that partner preference was so influential, this finding has been reported. In a study that measured a patient's and partner's desire for the patient to get a labor epidural, patients had higher desire scores [11]. This desire of partners for the patient to forego an epidural may be significant in some cases. Caregivers should be aware of this phenomenon in order to properly counsel patients and deal with the relationship dynamics that occur in the delivery unit. 

With univariate analysis we found that several socioeconomic factors were predictive of a patient not receiving an epidural. English as primary language, level of education, and type of insurance use were independent predictors of a patient receiving a labor epidural. Because these factors moved in parallel with partner preference, they did not add predictive power to the multivariate model. Other studies confirm our finding that socioeconomic factors are associated with epidural use [12–15]. One study by Hueston et al. [13] reviewed 8229 deliveries at five hospitals in the United States. They reported that epidural analgesia during labor was associated with the following: nulliparous, increasing maternal age, Caucasian ethnicity, and patients with private insurance. Sheiner et al. [14] found that higher education levels were associated with epidural use. Le Ray et al. [15] found that in France, higher epidural use was associated with the parturient cohabiting with their partner, being employed, professional/managerial occupation, and receiving adequate prenatal care. The French study is particularly enlightening since obstetric anesthesia care is free and universally available in France. Therefore, the data from Le Ray et al. is not confounded by the availability or affordability of obstetric anesthesia care.

Unlike the three studies mentioned above, our study took place within one maternity unit. On our service, socioeconomic factors should not influence access to an epidural because anesthesiologists at our institution have no financial incentives associated with performing epidurals. However, despite apparent equal and nonbiased access to labor epidurals at our institution, insurance, and ethnicity were associated with disparate epidural use. MediCal and Hispanic subgroups had significantly lower epidural usage than our insured and Caucasian subgroups. Why socioeconomic status is such a consistent predictor of epidural use (even in cases when issues of cost and access are eliminated) is unclear to us and warrants further study. 

Some hospital administrators may find it tempting to use the findings that certain socioeconomic groups utilize epidural analgesia to a lesser degree to justify low epidural rates in hospitals that service low income or predominantly minor ethnic populations. However, it should be clearly stated that the epidural rates in our study were still high in the MediCal and Hispanic subgroups (73% and 70%, resp.). In hospitals that service a low reimbursing population, very low epidural rates may be due to poorly staffed anesthesia services and a lack of access, not patient preference for a labor epidural. 

In our study and the Le Ray et al. [15] study, epidural usage rate was in the 75% to 80% range. This high epidural rate is in keeping with modern obstetric anesthesia trends in the US. Although epidural analgesia is a low-risk procedure, there are some risks associated with its use. It should be noted that of the women that did not get an epidural, a large number of these women desired natural childbirth as opposed to not being able to get their epidural or being afraid of possible complications. Maternity units with high rates of epidural may have abandoned non-pharmacological and more time consuming, less effective methods of dealing with labor pain. 

The sources of information used by women surveyed were very varied. This may reflect that no systematic method for labor analgesia information dissemination exists at our institution. The obstetric anesthesia service gives a labor analgesia presentation to expectant mothers twice a month, but only about 20% of our patient population attends. Our patients were most reliant on their obstetric physicians for labor analgesia information. Surprisingly, very few women used the Internet as a source of information. The Internet may become a more important source to learn about labor analgesic options in the near future. Further work is required to determine whether women desire more education about labor analgesic options before coming to the hospital [7, 9, 16]. Interestingly, Le Ray et al. [15] found that in France, where a prenatal visit with an anesthesia team member is required, they had approximately the same epidural rate as our hospital. It may be that when obstetric physicians are the primary source of labor analgesia education, epidural techniques are emphasized similarly to anesthesiologists.

In light of the fact that partner preference was associated with whether women received labor epidurals, educational efforts should be focused on the couple so that a consensus is reached before coming to the hospital. It is optimal that the partners have similar understandings and goals when they come in for labor and delivery. In addition, since significant portions of the women not receiving epidurals are Hispanic, and every effort should be made to communicate reliable information to this population group. Future studies should examine the impact of educational programs on labor epidural usage. 

A secondary intent of this study was to determine the efficiency of the obstetric anesthesia service at Lucile Packard Children's Hospital, Stanford, California. Based on the fact that everyone who expressed a strong desire for an epidural actually received one and that most people who did not get an epidural had an *a priori* desire not to have one, we are confident that we are delivering our service appropriately. One interesting observation is how many women changed their minds, from having a low desire for an epidural on arrival to the hospital to wanting one during the course of labor. This suggests that many women do not have clear expectations of the severity of labor pain and perhaps unable to make an informed choice until after they have actually experienced labor pain. Alternatively, our unit may use epidural analgesia as the default strategy to get women through difficult part of labor. More emphasis on non-pharmacological techniques may get a higher percentage of our parturients through labor without an epidural.

There were a number of limitations to this study. Due to logistical considerations, we obtained the information after delivery not immediately on admission to labor and delivery. Obviously recall bias could have influenced certain outcomes. Another potential limitation was that we asked the parturients for their perception of their partner's desire for them to have an epidural. It may have been more reliable to obtain this information directly from the partner. We elected this indirect approach due to the fact that the partners were not always available or present during the survey, and we considered that there would be a socioeconomic bias associated with which partners were available. In both cases, only doing pre-delivery surveys and asking the partners directly their opinions would have provided better data, but we would not been able to achieve the high (94%) capture of the parturients that was achieved in this study. In addition, there would have been considerable diurnal bias had we done the predelivery survey and asked the partners directly for their opinion, and we believe we may have less bias by only doing postdelivery surveys and asking the parturients for their partners' opinions.

In summary, our survey reflects that a significant number of women that do not want an epidural before labor end up receiving one during labor. Although we have not been able to develop a reliable predictive model to determine which women receive an epidural, we have identified a number of factors that may influence labor epidural use with partner preference and past experience being the two most important factors. This survey also confirmed the association of social economic and ethnic factors with epidural use.

## Figures and Tables

**Table 1:** Demographics, obstetric data as well as patient and partner desire for labor epidural.

	Received epidural (*n* = 243)	No epidural (*n* = 59)
Patients desire for epidural (0–100)	60 ± 40	11 ± 24*
Age (yr)	29 ± 7	26 ± 6
Height (cm)	163 ± 8	160 ± 3
Weight (kg)	72 ± 15	70 ± 15
BMI (gm/m^2^)	30 ± 5	30 ± 5
Wt of Baby (gm)	3308 ± 567	3266 ± 631
Nulliparous	52%	35%*
Previous epidural	81%	22%*
Pitocin used during labor	75%	37%*
Partner's preference for epidural		
Yes	61%	44%*
No	10%	16%
Undecided	29%	40%*
Language		
English	56%	29%*
Spanish	31%	66%*
Other	13%	5%
Did not finish high school	19%	46%*
Labor >6 hours	72%	32%*
MediCal Insurance	45%	69%*
Ethnicity		
Hispanic	42%	68%*
Caucasian	29%	7%*
East Asian	12%	10%
Indian Asian	8%	2%
Pacific Islander	6%	12%
African American	2%	2%
Other	2%	0%

Values expressed as mean ± SD, median(IQR) or percentages;

*P < .05 between groups that did and did not receive epidurals;

N.S. = No significant difference between the groups.

Data reported as percentages analyzed with Chi-square analysis. Other data analyzed using the Student *t*-test except the score of desiring an epidural. A Wilcoxon-Mann-Whitney U-test was used in this case since the two groups had differences in how the data were distributed.

**Table 2:** Multiple logistic regression analysis of factors associated with patients receiving epidural analgesia during labor.

Variable	Odds Ratio	95% CI	*P*-value
Partner preference			
Yes	25.1	5.2–122.0	<.0001
Undecided	11.4	22.9–45.6	.001
Prior epidural (yes/no)	9.0	2.5–32.5	.001
Language*	2.2	0.6–8.5	.264
Education^†^	0.9	0.6–1.4	.654
Insurance type**	0.7	0.1–4.0	.718
Age (years)^‡^	0.8	0.2–2.8	.752
Duration (hours)^††^	2.6	0.6–10.9	.192
Pitocin use (yes/no)	2.3	0.5–9.4	.263

Results were derived from a multiple logistic regression analyses comparing 243 patients who received a labor epidural to 59 patients who did not.

*Language (primary language English yes/no); ^†^Education (primary, junior high, high school, university, post graduate); **Insurance type (Medical/Private); ^‡^Age (≥ or <35 years); ^††^Duration (<6 or ≥6 hours).

**Table 3:** Responses to the question. “Before coming to the hospital, what was the *number one* reason why you might have wanted an epidural for labor?”

Number one reason	Received epidural (*n* = 243)	No epidural (*n* = 59)
Pain control	192 (79%)	38 (64%)
Previous experience	17 (7%)	2 (4%)
Encouraged by friend/family	15 (6%)	5 (8%)
Other	10 (4%)	8 (13%)
Relief of fatigue/stress	7 (3%)	4 (7%)
Encouraged by professional	2 (1%)	2 (4%)

Value expressed as number (%); *P* = NS between groups.

**Table 4:** Responses to the question. “Before coming to the hospital, what was the *number one* concern you had regarding epidurals, which may have led you towards avoiding an epidural for labor?”

Number one concern	Received epidural (*n* = 243)	No epidural (*n* = 59)
Concern of possible risks to themselves	131 (54%)*	14 (23%)
Afraid of delaying labor or increasing risk of cesarean	29 (12%)	10 (17%)
Desire for natural childbirth	27 (11%)*	16 (27%)
Concern over possible risk to baby	24 (10%)	6 (10%)
Pain from needle or procedure	22 (9%)	11 (19%)
Other	10 (4%)	2 (4%)

Value expressed as number (%); *P < .05 between groups that did and did not receive epidurals.

**Table 5:** Responses to the question. “After coming to the hospital, what was your main reason for *actually obtaining* an epidural for your labor?”

Main reason for obtaining a labor epidural	Received epidural (*n* = 242)
Pain control	211 (87%)
Relief of fatigue or stress	7 (3%)
Encouraged by friend or family	7 (3%)
Other	7 (3%)
Previous experience	5 (2%)
Encouraged by nurse	3 (1%)
Encouraged by obstetrician	2 (1%)

Value expressed as number (% of the total).

**Table 6:** Responses to the question. “After coming to the hospital, what was your main reason for *not obtaining* an epidural for labor pain?”

Main reason for not obtaining a labor epidural	No epidural received (*n* = 58)
Desire for natural childbirth	19 (33%)
Concern of possible risks to themselves	15 (26%)
Other	9 (16%)
Pain from needle or procedure	5 (9%)
Too far along in labor	5 (9%)
Afraid of delaying labor or increasing risk of cesarean	3 (5%)
Concern over possible risks to baby	2 (3%)

Value expressed as number (%).

## Appendix

### Survey Questions for All Subjects

**(1) Before coming to the hospital, how strongly (0–100 scale) did you desire an epidural for labor?** _______ (0 = not at all, 100 = absolutely)

**(2) Before coming to the hospital, what was the number one reason why you might have wanted an epidural for labor? (check only one item)**

1. pain control,
2. relief of fatigue/stress,
3. previous experience,
4. encouraged to obtain epidural by friend/family member,
5. encouraged to obtain epidural by OB, midwife, doula, labor educator…,
6. other, please list___________ .

**(3) Before coming to the hospital, what was the number one concern you had regarding epidurals, which may have led you towards avoiding an epidural for labor? (check only one item)**

1. concern over possible risks to me (back pain, headache, etc.),
2. concern over possible risks to baby,
3. pain from needle/procedure,
4. afraid of delaying labor or increasing the risk of c-section,
5. cost,
6. desire for natural childbirth,
7. discouraged by OB, midwife, doula, labor educator…,
8. other, please list_____________ .

**(4) Specific question depends on whether the patient received an epidural.**

**If the patient received an epidural: After coming to the hospital, what was your main reason for actually obtaining an epidural for your labor? (check only one item)**

1. pain control,
2. relief of fatigue/stress,
3. previous experience,
4. encouraged to obtain epidural by friend/family member,
5. encouraged to obtain by Obstetrician,
6. encouraged to obtain by Nurse,
7. other, please list___________ .

**If the patient did not receive an epidural: After coming to the hospital, what was your main reason for not obtaining an epidural for labor pain? (check only one item)**

1. concern over possible risks to me (back pain, headache, etc.),
2. concern over possible risks to baby,
3. afraid of pain from needle,
4. afraid of delaying labor or increasing the risk of c-section
5. cost,
6. desire for natural childbirth,
7. discouraged by OB or nurse,
8. too far along in labor,
9. medical contraindication,
10. anesthesiologist unavailable,
11. IV medication alone adequate,
12. needed a c-section,
13. other, please list_____________ .

**(5) What was your main source of information on epidurals prior to your labor? (check only one item)**

1. physician,
2. midwife,
3. family member/friend,
4. personal experience,
5. book/video/TV program,
6. childbirth class,
7. internet,
8. other, please list______________ .

**(6) Did your partner prefer you receive an epidural for labor? **

Yes / No / Neutral

**(7) Did anyone else in your family try to dissuade you from an epidural**?

Yes / No

If so, how is this person related to you? ____________________

**(9) Was this you first delivery?** Yes / No

**(10) If no, have you had a prior labor epidural?** Yes / No

**(11) How old are you?** ________yrs

**(12) How tall are you? ** ____ft___inches or ______cm

**(13) How much do you weigh? ** _______ lbs/kg (weight on admission)

**(14) What is your ethnicity**? Caucasian / Hispanic / East Asian / Asian Indian / African American / Pacific Islander / Other_______________________

**(15) What is the primary language you speak at home?**

English /Spanish / Other ________

**(16) What is your religion?** ______________

**(17) How much schooling have you completed?** Middle School / High School / College / Postgraduate / Elementary
